# Dr. W. H. Goddard

**Published:** 1883-04

**Authors:** 


Dr. W. H. Goddard.—Died at his home in Louisville, Ky., of chronic asthma, on Monday, March 5th, 1883, Dr. W. H. Goddard, in the seventy-fifth year of his age.

This announcement will fall like a great weight upon the hearts of many members of the dental profession. There are few men in practice who have not commenced their career since Dr. Goddard became a dentist. He had, with keen interest, watched the growth of the profession, and had the supreme satisfaction of living to see the few scattered members who made up the number in 1840, when he first opened an office, increase into a large and influential body of men, devoted to scientific research, with schools and a literature which eminently qualified them for membership in a learned profession.

For fifteen years Dr. Goddard was the faithful treasurer of the American Dental Association, and at its last meeting in Cincinnati he was with great unanimity elected its president ; the fitting climax of a long and useful professional life. There are few of those who were present and heard his inaugural remarks, who will not remember the impressive words then spoken, when he reminded the members that in the natural course of events, he could hardly hope to meet his brethren many more times. "Yet," said he, "I assume the gavel in the confidence of success ;" a confidence that was not misplaced, for he has succeeded in rounding out a long and useful life with a death at his post of honor, and he has left to his brethren the legacy of a conscientious and reputable professional life.

---

Vomiting in Phthisis.—To relieve this symptom, Dr. Woillez painted the pharynx with a solution of bromide of potassium, and found it very useful. A pencil of charpie dipped into a solution of pure bromine in two-thirds of water was passed rapidly into the pharynx before meals, the patient being required to abstain from expectoration after as long as possible. In several cases the vomiting was arrested by the first application, while in others the action, though less immediate, was beneficial.—*Journal de Thérapeutique.*

